# Chronic Kidney Disease in Children with Suspected Genetic Etiology: Diagnostic Yield and Clinical Implications

**DOI:** 10.3390/jpm16050232

**Published:** 2026-04-23

**Authors:** Aleksandra Paripović, Nikola Ilić, Marina Perić, Slavica Ostojić, Danijela Radivojević, Luka Nikolić, Adrijan Sarajlija

**Affiliations:** 1 Department of Nephrology, Mother and Child Health Care Institute of Serbia “Dr Vukan Cupic”, 11070 Belgrade, Serbia; aleksandra.paripovic@imd.org.rs (A.P.); marina.peric@imd.org.rs (M.P.); luka.nikolic@imd.org.rs (L.N.); 2 Department of Pediatrics, Faculty of Medicine, University of Belgrade, 11000 Belgrade, Serbia; slavica.ostojic@imd.org.rs (S.O.); adrijan.sarajlija@imd.org.rs (A.S.); 3 Division of Clinical Genetics, Mother and Child Health Care Institute of Serbia “Dr Vukan Cupic”, 11070 Belgrade, Serbia; 4 Department of Neurology, Mother and Child Health Care Institute of Serbia “Dr Vukan Cupic”, 11070 Belgrade, Serbia; 5 Laboratory of Medical Genetics, Mother and Child Health Care Institute of Serbia “Dr Vukan Cupic”, 11070 Belgrade, Serbia; danijela.radivojevic@imd.org.rs

**Keywords:** chronic kidney disease, pediatrics, genetic diagnosis, next-generation sequencing, copy number variants, extrarenal manifestations, personalized medicine, precision nephrology

## Abstract

**Background:** Chronic kidney disease (CKD) in children is frequently associated with underlying genetic etiologies, particularly in cases with early onset, congenital anomalies, or multisystem involvement. The integration of molecular diagnostics into routine nephrological practice represents an important step toward personalized medicine in pediatric CKD. **Methods:** This retrospective observational study included 50 pediatric patients with CKD stages 2–5 and suspected hereditary etiology evaluated at a tertiary pediatric center. All patients underwent genetic testing using next-generation sequencing and/or chromosomal microarray analysis. Clinical characteristics, CKD stage, extrarenal manifestations, and disease progression were analyzed in relation to genetic findings. Associations between clinical variables and genetic diagnosis were assessed using appropriate statistical tests, including multivariable logistic regression. **Results:** A positive genetic diagnosis was identified in 28 patients (56%), including 21 monogenic disorders detected by next-generation sequencing and 7 pathogenic copy number variants identified by chromosomal microarray analysis. Extrarenal manifestations were present in 48% of patients and were significantly associated with a higher diagnostic yield (75% vs. 42.3%; OR = 4.09; 95% CI: 1.23–13.61; *p* = 0.007). Psychomotor delay was strongly associated with pathogenic copy number variants (*p* < 0.001). Patients with confirmed genetic etiologies exhibited significantly higher rates of CKD progression compared with genetically negative individuals (82.1% vs. 22.7%; OR = 15.64; 95% CI: 3.90–62.7; *p* < 0.001). In multivariable analysis, genetic diagnosis shows association with disease progression after adjustment for age and baseline renal function. **Conclusions:** Genetic testing provided a molecular diagnosis in more than half of children with CKD and suspected hereditary etiology. Extrarenal manifestations were strongly associated with a higher diagnostic yield, while confirmed genetic etiologies may be associated with CKD progression. These findings support the early integration of genetic diagnostics into the evaluation of pediatric CKD to improve prognostic assessment and enable more personalized management strategies.

## 1. Introduction

Chronic kidney disease (CKD) in children represents a significant cause of long-term morbidity and mortality, with lifelong implications for growth, cardiovascular health, and quality of life [1,2]. Based on population-based studies and registry data, the prevalence of chronic kidney disease in children has been estimated at approximately 30–100 cases per million age-related population (about 0.003–0.01%), although early stages remain underdiagnosed [3]. Unlike adult CKD, which is most commonly caused by acquired conditions such as diabetes and hypertension, pediatric CKD is predominantly driven by congenital anomalies of the kidney and urinary tract (CAKUT) and hereditary nephropathies [3,4]. The most common causes of CKD in children in the United States are congenital anomalies of the kidney and urinary tract (CAKUT), steroid-resistant nephrotic syndrome (SRNS), chronic glomerulonephritis, and ciliopathies, which together account for more than 70% of cases [4]. To date, 600 genes associated with CKD have been identified, accounting for 50% of CKD cases in pediatric cohorts and 20% in the adult population [4,5,6]. Approximately 30% of children have a monogenic cause [7,8,9].

Over the past decade, advances in genomic technologies, particularly next-generation sequencing (NGS) and chromosomal microarray analysis (CMA), have substantially improved the diagnostic yield in children with suspected hereditary kidney disease [10,11,12]. Studies have reported molecular diagnostic rates ranging from 20% to over 50%, depending on patient selection criteria, age at presentation, and the presence of extrarenal features [13,14,15]. The identification of a genetic etiology may provide critical information for diagnosis, prognosis, therapeutic decision-making, and family counseling, thereby aligning pediatric nephrology with the broader paradigm of personalized medicine [16,17].

However, several important questions remain unresolved. First, the optimal selection criteria for genetic testing in pediatric CKD remain debated. While some authors advocate broad genomic testing in most children with early-onset CKD, others propose more targeted approaches based on specific clinical features, such as family history or syndromic presentation [11,18]. Second, the prognostic implications of a confirmed genetic diagnosis are not fully established. Although some studies suggest that genetic etiologies may be associated with distinct clinical trajectories, including earlier progression to end-stage kidney disease, other reports have not demonstrated a consistent or independent association. Notably, it has been emphasized that translation of genetic findings into better outcomes requires longitudinal follow up of large cohorts, highlighting the current limitations of available evidence and the lack of robust prognostic data [19]. This inconsistency likely reflects substantial genetic and phenotypic heterogeneity, as well as ongoing challenges in variant interpretation, including the risk of misclassification and interobserver variability. Overall, further well-designed longitudinal studies are needed to clarify the true prognostic value of genetic findings [19,20,21].

Another area of ongoing discussion concerns the clinical significance of extrarenal manifestations. Multisystem involvement, including neurodevelopmental delay, cardiovascular anomalies, and dysmorphic features, has been associated with higher diagnostic yields, particularly in cases involving copy number variants [15,22]. However, the extent to which these features should influence genetic testing strategies and clinical management remains incompletely defined.

In this context, real-world data from tertiary pediatric nephrology centers are of great importance for refining clinical indications for genetic testing and for understanding the prognostic impact of molecular diagnoses. Such data are particularly valuable in regions where access to advanced genetic diagnostics has only recently become more widely available.

Recent proposals for step-by-step multidisciplinary diagnostic strategies further emphasize the importance of selecting the appropriate genetic test based on clinical presentation and associated features [23,24].

The aim of this study was to evaluate the diagnostic yield and clinical correlates of genetic testing in a cohort of children with CKD and suspected hereditary etiology, and to assess the relationship between genetic diagnosis, extrarenal manifestations, and disease progression.

## 2. Materials and Methods

### 2.1. Study Design and Setting

This retrospective observational study was conducted at the Department of Nephrology, Mother and Child Health Care Institute of Serbia “Dr Vukan Čupić”, Belgrade, Serbia. The study included pediatric patients evaluated for CKD with suspected genetic etiology over the period from 2020 to 2025. Genetic testing was indicated in pediatric patients with CKD stage II–V, encompassing the following groups: patients with CAKUT, patients with associated extrarenal manifestations, patients with nonimmune glomerulopathies, patients with tubulopathies or patients with renal ciliopathies. Patients with CKD stage II–V were evaluated by a pediatric nephrologist, and those with features suggestive of hereditary kidney disease were referred for clinical genetics consultation. Genetic testing was selected based on clinical assessment, and results were interpreted in a multidisciplinary setting to establish the final diagnosis.

The research was performed in accordance with the principles of the Declaration of Helsinki. Approval for the study was obtained from the institutional ethics committee. Due to the retrospective nature of the study and the use of anonymized clinical data, informed consent requirements were waived in accordance with local regulations.

### 2.2. Study Population

The study cohort consisted of 50 pediatric patients (aged from 1 month to 18 years) who were genetically evaluated due to CKD with suspected hereditary or syndromic background. Patients were included if they met the following criteria: confirmed diagnosis of CKD based on clinical and laboratory findings; suspicion of genetic etiology based on clinical presentation, family history, or presence of congenital anomalies; and availability of genetic testing results.

Patients who did not undergo genetic testing were excluded from the analysis and patients with insufficient clinical data were excluded from the study.

### 2.3. Clinical Data Collection

Clinical and demographic data were obtained retrospectively from medical records. The following variables were collected: sex, age at diagnosis, age at genetic testing, CKD stage at diagnosis and at the most recent follow-up, presence and type of extrarenal manifestations, presence of psychomotor delay, presence and type of congenital anomalies.

CKD staging was performed according to the KDIGO pediatric chronic kidney disease classification. Glomerular filtration rate (GFR) was estimated using the modified Schwartz formula [25,26]. Rapid decline in kidney function was defined in accordance with KDIGO guidelines as a decrease in estimated glomerular filtration rate (eGFR) of ≥5 mL/min/1.73 m^2^ per year, reflecting clinically significant progression of chronic kidney disease.

Extrarenal manifestations were defined as involvement of organ systems outside the kidneys, including cardiovascular, central nervous system, skeletal, endocrine, auditory, or dysmorphic features.

### 2.4. Genetic Testing

All patients with CKD and suspicion of underlying genetic condition underwent genetic evaluation as part of their clinical diagnostic workup during the observed period. The applied genetic methods included:

- Whole-exome sequencing;
- Chromosomal microarray analysis (CMA) for the detection of pathogenic copy number variants (CNVs).

The selection of the first-tier genetic test was based on the patient’s clinical phenotype. WES was performed as a first-tier test in 47/50 patients as the principal diagnostic modality for detecting monogenic causes. CMA was conducted in 10 patients, either as a first-line test in cases with a high suspicion of chromosomal imbalance (*n* = 3) or as a second-line investigation following negative WES results (*n* = 7).

Overall, while the majority of patients underwent WES-based evaluation, CMA was selectively applied based on phenotypic features suggestive of genomic imbalance. Thus, although not all patients underwent identical first-line testing, the diagnostic approach followed a phenotype-driven strategy consistent with contemporary clinical practice.

Genetic findings were classified according to the American College of Medical Genetics and Genomics (ACMG) guidelines. A positive genetic diagnosis was defined as the identification of a pathogenic or likely pathogenic variant consistent with the patient’s phenotype. Cases without such findings were classified as genetically negative. Genetically negative cases were subjected to periodic reanalysis in accordance with updated ACMG variant classification guidelines.

### 2.5. Outcome Measures

The primary outcome of the study was the diagnostic yield of genetic testing, defined as the proportion of patients in whom a pathogenic or likely pathogenic variant explaining the renal phenotype was identified.

Secondary outcomes included the distribution of genetic etiologies within the cohort, the association between clinical and demographic characteristics and the probability of a positive genetic diagnosis, the relationship between extrarenal manifestations and genetic diagnostic yield, and the association between genetic etiology and progression of chronic kidney disease (CKD).

### 2.6. Statistical Analysis

Statistical analyses were performed using appropriate descriptive and inferential methods. Continuous variables were assessed for distribution and reported as medians with interquartile ranges (IQRs), while categorical variables were expressed as counts and percentages. Group comparisons were performed using the Mann–Whitney U test for continuous variables and the Pearson chi-square test or Fisher’s exact test, as appropriate, for categorical variables. Effect sizes for contingency tables were estimated using Cramer’s V, where applicable.

To evaluate factors associated with CKD progression, multivariable logistic regression analysis was performed. Variables included in the model were selected a priori based on clinical relevance and comprised genetic diagnosis status, age at diagnosis, and baseline renal function (estimated glomerular filtration rate). Adjusted odds ratios (aORs) with 95% confidence intervals (CIs) were calculated. Given the relatively small sample size, the number of variables in the model was limited to reduce the risk of overfitting, and the results should be interpreted with caution. All statistical analyses were conducted using IBM SPSS Statistics software (Version 25.0, IBM Corp., Armonk, NY, USA).

## 3. Results

The cohort comprised 24 male (48%) and 26 female (52%) patients evaluated for CKD with suspected genetic etiology, ranging in age from 1 month to 18 years. A positive genetic diagnosis was established in 28 patients (56%), while 22 (44%) had negative genetic findings. Regarding the diagnostic modality, monogenic disorders identified by next-generation sequencing accounted for 42% of cases and copy number variants detected by chromosomal microarray analysis for 14%, whereas 44% of patients had normal genetic results. CMA identified pathogenic copy number variants in 4 cases in which prior WES was non-diagnostic. Notably, all patients with CMA-detected abnormalities exhibited extrarenal manifestations.

At the time of genetic evaluation, the majority of patients were classified as CKD stage 2 (46%), while advanced disease (CKD stages 3–4) was observed in only 8% of cases. The median follow-up time was 4.0 years (interquartile range 6 years), with a mean of 5.52 ± 3.97 years (range 1–16 years). During the follow-up period, disease progression from one stage to another occurred in 28 of 50 patients (56%). At the most recent clinical follow-up, the majority of patients were classified as CKD stage 2 (68%), while 16% were in stage 3, 14% in stage 4 and one patient (2%) in stage 5.

The median age at clinical diagnosis was 3 months (IQR 11), whereas the median age at performing genetic diagnostics was 45 months (IQR 99). Both variables demonstrated marked right-skewed distributions, supporting the use of median values for descriptive reporting.

Patients with negative genetic results had a median age at genetic testing of 48 months (IQR 84), compared with 36 months (IQR 108) in those with a confirmed genetic diagnosis. Genetic analyses tended to be performed earlier in patients who ultimately received a molecular diagnosis, although substantial variability in testing age was observed in both groups. There was no significant difference in the age at genetic testing between patients with positive and negative genetic results (median 36 vs. 48 months; Mann–Whitney U = 293, Z = −0.23, *p* = 0.819).

A positive genetic diagnosis was established in 28 patients (56%), including 21 cases of monogenic disorders detected by next-generation sequencing (42%) and 7 pathogenic copy number variants identified by chromosomal microarray analysis (14%). Normal genetic findings were observed in 22 patients (44%). The etiologies identified by NGS were highly heterogeneous, encompassing ciliopathies (5/21), glomerulopathy (5/21), CAKUT (11/21) and nepholithiasis (1/21). One patient was found to carry pathogenic variants in both the *NPHP3* and *HNF1B* genes. All patients with pathogenic copy number variants (7/28) had CAKUT (Table 1).

**Table 1:** Identified genetic etiologies in the study cohort (gene-based classification) (*n* = 50).

Genetic Category	Gene/Genomic Alteration	*n* (%)
Normal genetic result	No pathogenic variant detected	22 (44.0)
Recurrent monogenic finding (NGS)	*HNF1B*	6 (12.0)
Single-gene findings (each identified in one patient)	*HPSE2*, *SALL1*, *TSC1*, *SMARCAL1*, *IFT140*, *TARS2*, *ATP6V0A4*, *ADCK4*, *NPHP1*, *NPHS1*, *NUP93*, *PKD1*, *ELN*, *EYA1*, *PKHD1*, *GATA3*	15 (32.0)

A positive genetic diagnosis was identified in 12 of 24 male patients (50%) and in 16 of 26 female patients (61.5%). There was no statistically significant association between patient sex and the likelihood of a positive genetic diagnosis (Pearson χ^2^ = 1.21, df = 1, *p* = 0.271). This result remained non-significant following continuity correction (*p* = 0.415) and Fisher’s exact test (two-sided *p* = 0.390). Female patients showed a higher, but non-significant, probability of a positive genetic diagnosis compared with male patients (odds ratio [OR] = 1.89; 95% confidence interval [CI]: 0.64–5.58).

Extrarenal manifestations were present in 24 of 50 patients (48%). The most frequently observed features included cardiovascular and central nervous system involvement (each 18%), followed by psychomotor delay (12%). Structural extrarenal congenital anomalies were present in 30% of cases. The distribution of extrarenal clinical features observed in the study cohort is summarized in Table 2.

A genetic diagnosis was established in 18 patients (75%) with extrarenal manifestations, whereas 6 patients (25.0%) had negative genetic findings. Among the 26 patients without extrarenal manifestations, 11 (42.3%) had a positive genetic diagnosis and 15 (57.7%) were genetically negative. A significant association was observed between the presence of extrarenal manifestations (ER) and genetic diagnostic outcomes (Pearson’s χ^2^ = 9.89, df = 2, *p* = 0.007; Cramer’s V = 0.45), indicating a moderate-to-strong effect size. Patients with extrarenal manifestations had a significantly higher likelihood of receiving a positive genetic diagnosis compared with those without such manifestations (odds ratio [OR] = 4.09; 95% confidence interval [CI]: 1.23–13.61), indicating an approximately fourfold increase in diagnostic yield.

Patients with normal genetic findings predominantly lacked extrarenal features (15/22), whereas those with monogenic disorders identified by next-generation sequencing showed a balanced distribution of extrarenal involvement (10/21 with manifestations apart from kidney). Notably, all patients with copy number variants detected by chromosomal microarray analysis exhibited extrarenal manifestations (7/7), highlighting a strong association between multisystem phenotypes and structural genomic alterations.

Psychomotor delay was significantly associated with genetic diagnostic group (Pearson’s χ^2^ = 15.71, df = 2, *p* < 0.001; Cramer’s V = 0.56), representing a strong effect size. Namely, developmental delay was rare among patients with normal genetic results (1/22) and those with monogenic disorders (1/21) but was markedly prevalent in the CMA-positive group (4/7). This finding indicates a substantial presence of neurodevelopmental impairment among CKD patients harboring pathogenic copy number variants.

Rapid CKD progression, defined according to KDIGO criteria for accelerated eGFR decline, was significantly more frequent among patients with a genetically confirmed diagnosis compared to those without. This association was supported by Pearson’s chi-square test (χ^2^ = 7.229, *p* = 0.007) and confirmed by Fisher’s exact test (two-sided *p* = 0.011). Patients with a molecularly established etiology exhibited substantially increased odds of rapid progression, with an approximately 7.7-fold higher risk compared to genetically negative cases (OR = 7.72, 95% CI: 1.56–38.2).

Multivariable logistic regression analysis demonstrated that a confirmed genetic diagnosis remained independently associated with CKD progression after adjustment for age and baseline renal function (adjusted odds ratio [aOR] = 19.43, 95% confidence interval [CI]: 1.58–238.9, *p* = 0.019). Baseline renal function was also a significant predictor of disease progression (aOR = 0.009, 95% CI: 0.001–0.12, *p* = 0.002), whereas age at diagnosis was not independently associated with progression risk (aOR = 1.01, 95% CI: 0.99–1.04, *p* = 0.213). The overall model demonstrated strong predictive performance (Nagelkerke R^2^ = 0.752) with an overall classification accuracy of 88%.

A statistically significant association was identified between CAKUT phenotype and genetic diagnostic category (Pearson’s χ^2^ = 11.13, df = 4, *p* = 0.025; Fisher’s exact test *p* = 0.014). In patients with monogenic disorders detected by next-generation sequencing, 30% had CAKUT and 70% did not. All copy number variants identified by chromosomal microarray analysis were confined to patients with CAKUT, namely renal hypodysplasia. However, the renal hypodysplasia group demonstrated heterogeneous genetic outcomes, including monogenic diagnoses in 26.7% of cases, copy number variants in 23.3% and normal genetic results in 49.6%. Renal agenesis was predominantly associated with negative genetic findings (66.7%).

## 4. Discussion

In this study, we evaluated the diagnostic yield and clinical correlates of genetic testing in a cohort of children with chronic kidney disease stage 2–5 (CKD) and suspected hereditary etiology. A molecular diagnosis was established in more than half of the patients, and confirmed genetic etiologies were strongly associated with extrarenal manifestations and disease progression.

### 4.1. Diagnostic Yield of Genetic Testing in Pediatric CKD

The overall molecular diagnostic rate of 56% observed in our cohort is at the higher end of the range reported in previous studies, which typically describe yields between 20% and 50%, depending on patient selection criteria and testing strategies. This relatively high diagnostic yield likely reflects careful clinical selection of patients with suspected hereditary or syndromic CKD. It also highlights the value of combining next-generation sequencing with chromosomal microarray analysis, particularly in patients with complex phenotypes [6,21]. We observed a higher percentage of CKD pediatric patients with a positive genetic finding in comparison to the studies conducted in adult cohorts, especially if CAKUT was present [17].

Monogenic disorders accounted for the majority of positive findings, while copy number variants represented a smaller but clinically specific subgroup of cases. This distribution is consistent with prior reports showing that single-gene disorders predominate in early-onset CKD, whereas structural genomic alterations are more commonly associated with syndromic presentations and neurodevelopmental involvement.

The distribution of different kidney diseases in our group of pediatric CKD patients is heterogeneous and includes ciliopathies, glomerulopathies, and CAKUT, being consistent with previously described pediatric cohorts [27,28].

In our study, the majority of patients carried a heterozygous mutation in *HNF1B*. According to most published studies, the most common genetic causes of CAKUT are mutations in the *HNF1B* or *PAX2* genes [29,30,31,32].

In a prospective cohort study (CkiD) conducted in North America that included children with CKD, without clinically detectable syndromic disease, chromosomal microarray analysis identified pathogenic copy number variants in 7.4% of patients [22]. In our study cohort, this proportion was nearly twofold higher. However, all of our patients with CNV had extrarenal manifestations. Other authors reported low yield of chromosomal microarray when performed after targeted exome sequencing in pediatric patients [31].

### 4.2. Significance of Extrarenal Manifestations

One of the most important findings of this study was the strong association between extrarenal manifestations and a positive genetic diagnosis. Patients with multisystem involvement had approximately a fourfold higher likelihood of receiving a molecular diagnosis compared with those with isolated renal disease. This observation is in line with previous studies demonstrating that syndromic features significantly increase the diagnostic yield of genetic testing [20,22,27].

Notably, all patients with pathogenic copy number variants exhibited extrarenal manifestations, and psychomotor delay was particularly present in this group. This finding reinforces the concept that neurodevelopmental impairment and structural congenital anomalies should prompt consideration of chromosomal microarray analysis in children with CKD. From a clinical perspective, the presence of extrarenal features may serve as an important indicator for prioritizing comprehensive genetic testing.

In the study by Chen et al., extrarenal manifestations were present in 28.7% of CKD patients, with yield of positive genetic diagnosis of 42.7% in this subpopulation, which is lower compared to our results [27]. However, patients with extrarenal manifestations in aforementioned cohort were more likely to be diagnosed by molecular genetic testing, which is consistent with our findings. Prevalence of extrarenal manifestations in our study was higher (48%) in comparison to other pediatric CKD cohorts (14,1 do 39%). The most prevalent extrarenal manifestations in these studies were cardiovascular anomalies which are consistent with our results [6,29].

Our findings are consistent with previous studies demonstrating that the presence of extrarenal manifestations significantly increases the likelihood of identifying a monogenic cause of CKD, particularly in pediatric cohorts [17,20,27,31]. High prevalence of neurodevelopmental disorders in children with CKD carrying pathogenic copy number variants is similar in our and previous studies [22].

### 4.3. Genetic Etiology and Disease Progression

Another key observation was the higher rate of CKD progression among patients with confirmed genetic etiologies. Children with a genetic cause of CKD had increased odds of disease progression compared with genetically negative patients. This finding suggests that genetic causes of CKD may be associated with more rapid disease trajectories as already suggested by other authors; however, this observation reflects an association rather than a causal relationship, given the retrospective design of the study [27]. In our study, multivariate regression analysis demonstrated that a positive genetic finding in CKD patients represents a risk factor for disease progression, with initial CKD stage acting as additional independent predictor of rapid disease progression. These findings suggest an association between genetic diagnosis and more rapid CKD progression; however, the wide confidence interval indicates residual uncertainty, and the results should be interpreted with caution pending validation in larger cohorts.

This observation is consistent with previous studies demonstrating that the underlying etiology of CKD significantly influences the rate of disease progression, particularly in hereditary kidney disorders [33]. More precisely, mutations in genes associated with glomerular or developmental kidney disorders, such as *NPHS2*, *NPHS3*, and *WT1*, are typically associated with an earlier onset of renal insufficiency. In contrast, variants in genes such as *NPHP1* or *PAX2* are more frequently linked to a later development of chronic kidney disease [27]. These reports support the biological plausibility of the clinical course observed in our cohort. This aligns well with emerging evidence that molecular diagnoses can provide prognostic information in CKD beyond traditional clinical and laboratory parameters [34]. In certain genetic kidney diseases, the natural history is largely determined by the underlying molecular defect, making genotype-phenotype correlation crucially informative for risk stratification and long-term management [20].

From the perspective of personalized medicine, the identification of a specific genetic etiology enables more accurate prognostic counseling, tailored surveillance strategies, and, in some cases, targeted therapeutic approaches. For example, coenzyme Q10 supplementation has been reported to improve outcomes in patients with mitochondrial nephropathy caused by *ADCK4* mutations [35]. Moreover, establishing a molecular diagnosis can help avoid unnecessary immunosuppressive therapy and invasive procedures such as kidney biopsy, while also informing clinical management and planning for kidney transplantation [36]. Furthermore, genetic diagnoses have important implications for family screening and reproductive counseling [37].

### 4.4. Implications for Personalized Approach in Pediatric Nephrology

Genetic diagnostics has become an important component of clinical diagnostic workflow in pediatric nephrology. Rather than relying solely on phenotypic classification, molecular diagnoses allow for genotype-driven disease categorization, which may better reflect underlying pathophysiological mechanisms.

In our cohort, the high diagnostic yield and the observed association between genetic etiologies and disease progression support the consideration of early genetic testing as an important component of the diagnostic workflow in children with CKD, particularly in those with early onset or extrarenal manifestations. However, given the retrospective design and the selected nature of the cohort, these findings should be interpreted with caution. Prospective studies in broader populations are needed to further define the role and optimal timing of genetic testing in this setting. Nevertheless, such an approach is consistent with the principles of personalized medicine, in which diagnostic and therapeutic decisions are guided by the individual patient’s molecular profile [27,36].

All patients in our cohort who underwent chromosomal microarray analysis presented with CAKUT and additional clinical elements, particularly neurodevelopmental delay. Therefore, in patients with renal disease and confirmed pathogenic copy number variants, even in the absence of a syndromic presentation, close monitoring of neurodevelopmental progress is recommended [22].

### 4.5. Potential of Early or Preventive Genetic Screening Strategies

Beyond its diagnostic value, the role of earlier genetic testing in pediatric CKD warrants consideration within a personalized medicine framework. In selected high-risk populations, such as patients with prenatally detected CAKUT or a positive family history, early genetic evaluation may enable improved risk stratification and anticipatory monitoring [38]. In this context, genetic diagnosis could potentially identify patients at increased risk of rapid progression, as suggested by our findings, and imply more intense follow-up and genotype-driven therapeutic decision-making. However, the implementation of broader screening strategies, including neonatal or population-based approaches, remains challenging. Limitations include variable penetrance and expressivity of many genetic variants, uncertainty in the interpretation of variants of unknown significance, and the current lack of genotype-specific interventions for many conditions. In addition, ethical considerations—particularly in pediatric populations—must be carefully addressed, including issues related to informed consent, the psychosocial impact of early diagnosis, and the management of incidental findings [39]. Therefore, while early genetic testing holds promise, its application should remain targeted and clinically guided, pending further evidence from prospective studies. Future studies should evaluate the cost-effectiveness and long-term clinical impact of early genetic screening strategies in pediatric CKD.

### 4.6. Study Limitations

Several important limitations of this study should be emphasized. First, the retrospective design introduces potential selection bias, as genetic testing was performed based on clinical indication rather than well-established algorithm.

Secondly, the study cohort represents a selected population from a tertiary referral center, which may limit generalizability. Therefore, the observed diagnostic yield may be overestimated due to selection bias inherent to a highly distilled cohort of patients referred to a tertiary care center for suspected hereditary CKD.

Furthermore, the relatively small sample size, combined with the inclusion of multiple variables in the regression model, raises the possibility of model overfitting, particularly in exploratory analyses.

Finally, functional validation of identified variants was not performed, and variant interpretation relied on established clinical classification frameworks. These limitations highlight the need for prospective, multicenter studies to validate the findings and further clarify clinical benefit of genetic testing in pediatric CKD.

### 4.7. Future Directions

Future prospective, multicenter studies with larger cohorts are needed to better define the prognostic impact of specific genetic etiologies in pediatric CKD. Longitudinal studies integrating genomic, clinical, and biomarker data may further refine risk stratification models and support the development of targeted therapies.

The increasing availability of comprehensive genomic testing also raises the possibility of earlier diagnosis, including in presymptomatic stages, which could significantly influence long-term outcomes and management strategies.

## 5. Conclusions

Genetic testing provided a molecular diagnosis in more than half of children with CKD and suspected hereditary etiology in our cohort, confirming its high diagnostic value in pediatric nephrology practice. The presence of extrarenal manifestations was associated with a higher likelihood of identifying a genetic cause, particularly in patients with pathogenic copy number variants. Importantly, a confirmed genetic diagnosis may be independently associated with CKD progression, suggesting that molecular findings may contribute not only to etiological clarification but also to prognostic stratification.

These results support the early integration of genetic testing into the diagnostic workflow of children with CKD, especially in those with early disease onset, CAKUT, or multisystem involvement. Beyond establishing the cause of disease, molecular diagnosis may improve clinical decision-making, guide follow-up and family counseling, and potentially facilitate a more personalized approach to management.

## Figures and Tables

**Table 2:** Frequency of clinical and extrarenal features in the study cohort (*n* = 50).

Variable	Present *n* (%)	Absent *n* (%)
Extrarenal manifestations (overall)	24 (48.0)	26 (52.0)
Cardiovascular involvement	9 (18.0)	41 (82.0)
Central nervous system involvement	9 (18.0)	41 (82.0)
Skeletal abnormalities	3 (6.0)	47 (94.0)
Dysmorphic facial features	3 (6.0)	47 (94.0)
Short stature/growth impairment	2 (4.0)	48 (96.0)
Psychomotor delay	6 (12.0)	44 (88.0)
Epilepsy	3 (6.0)	47 (94.0)
Hormonal abnormalities	3 (6.0)	47 (94.0)
Extrarenal structural congenital anomalies	15 (30.0)	35 (70.0)
Hearing impairment	3 (6.0)	47 (94.0)

## Data Availability

The data presented in this study are only available on request from the corresponding author due to privacy or ethical restrictions.
